# Age-Dependent Variation of Lamina Cribrosa Displacement During the Standardized Valsalva Maneuver

**DOI:** 10.1038/s41598-019-43206-6

**Published:** 2019-04-30

**Authors:** Yong Woo Kim, Dong Hyun Lee, Hyung Bin Lim, Baek-Lok Oh, Young Kook Kim, Michael J. A. Girard, Jean Martial Mari, Ki Ho Park, Jin Wook Jeoung

**Affiliations:** 1 Department of Ophthalmology, Seoul National University Hospital, Seoul National University College of Medicine, Seoul, Korea; 2 Department of Ophthalmology, Armed Forces Capital Hospital, Seongnam, Korea; 3 Department of Biomedical Engineering, National University of Singapore, Singapore, Singapore; 4 Singapore Eye Research Institute, Singapore, Singapore; 5 University of French Polynesia, Tahiti, French Polynesia

**Keywords:** Eye manifestations, Medical imaging

## Abstract

Based on biomechanical theory, lamina cribrosa (LC) displacement, the key component of progressive glaucomatous change, is presumed to be dependent on intraocular pressure (IOP) as well as tissue stiffness of LC. In the performance of the Valsalva maneuver, both IOP and cerebrospinal fluid pressure can increase. The present study investigated the age-dependent variation of LC displacement during the standardized Valsalva maneuver in healthy subjects. Sixty-three (63) eyes (age range: 20–76 years) were prospectively underwent IOP measurement and Cirrus HD-OCT optic disc scans before and during the standardized Valsalva maneuver. During the standardized Valsalva maneuver, the IOP significantly increased from 13.2 ± 2.9 mmHg to 18.6 ± 5.2 mmHg (*P* < 0.001). The maximal LC depth significantly decreased in the younger age groups (age: 20 s to 40 s) but not in the older age groups (age: over 50). The BMO distance did not change significantly. Younger age (*P* = 0.009), a smaller increase of IOP during the Valsalva maneuver (*P* = 0.002), and greater baseline maximal LC depth (*P* = 0.013) were associated with more anterior displacement of the LC during the standardized Valsalva maneuver. Taken together, age as well as translaminar pressure dynamics seems to play a crucial role in LC biomechanics.

## Introduction

Posterior deformation of the lamina cribrosa (LC) is one of the principal components of progressive optic nerve head (ONH) change in glaucoma^1^. Experimental animal studies (monkey eyes) have confirmed posterior LC displacement in cases of chronic laser-induced intraocular pressure (IOP) elevation^2,3^. This phenomenon coincides with scleral canal expansion and posterior bowing of the peripapillary sclera as well as the loss of retinal nerve fiber layer (RNFL) tissue and LC remodelling^4,5^. Evidence from *in vivo* human optical coherence tomography (OCT) imaging studies is growing that LC depth and curvature are increased in glaucomatous eyes^6–9^, which happens in the early stages of glaucoma^10^. In the biomechanical perspective, finite element models predict that ONH deformation depends on IOP as well as tissue properties (stiffness and compliance) of the LC and sclera^11–14^.

Postmortem human-donor eye studies have revealed that the tissue properties of the LC and sclera differ by age and race^15^. The peripapillary sclera stiffens with age, which effect is more accelerated in eyes of African descent compared with European^16^. This can lead to age-driven loss of scleral compliance in aged and African-descent eyes and, thus might have increased susceptibility to glaucoma. The role of tissue properties in IOP-related LC deformation is further evidenced by racial differences of LC behavior on acute IOP elevation. Fazio *et al*.^17^ demonstrated different LC depth changes between European and African eyes in response to acute IOP elevation, the latter showing greater posterior bowing of the LC.

Cerebrospinal fluid pressure (CSFP) has been proposed to be another crucial component of LC displacement^18^. CSFP have shown to be decreased in normal-tension glaucoma (NTG) eyes, the consequence being an increased pressure difference between IOP and CSFP (i.e., TLPD, translaminar pressure difference). This finding has been posited as a possible factor in the pathogenesis of NTG^19,20^. However, recent prospective study revealed no evidence of a decrease in the intracranial pressure of NTG patients, leaving the role of CSFP in glaucoma inconclusive^21^. Despite controversies to linkage between CSFP and glaucoma development, the position of LC, representatively measured as LC depth, can be dependent on translaminar pressure dynamics^22^. This phenomenon has been further evidenced by posterior displacement of the LC in pediatric patients with surgical decompression due to increased intracranial pressure^23^. However, recent study with numerical simulated model exhibited less remarkable influence of CSFP than IOP on ONH biomechanics, and proposed different role of IOP and CSFP on ONH deformation^24^. In this regard, further investigation and better understanding for LC behavior according to the translaminar pressure dynamics is imperative.

In the performance of the Valsalva maneuver, both IOP and CSFP can increase. Zhang *et al*.^25^ investigated this effect in subjects with lumbar puncture, and confirmed that the CSFP increment exceeded that of IOP, with the result that the TLPD may have been decreased or reversed. Our group recently demonstrated anterior displacement of the LC during the standardized Valsalva maneuver in young healthy eyes^26^. Our subsequent study showed that the Valsalva-induced anterior LC displacement was prominent in healthy eyes but not in glaucomatous eyes^27^. Having hypothesized that age-related change of the LC and peripapillary sclera influences the degree of LC displacement during the standardized Valsalva maneuver, we investigated, in the present study, whether LC depth change during the standardized Valsalva maneuver differed according to subject age and evaluated the factors associated with LC displacement.

## Methods

The present study was approved by the Armed Forces Medical Command Institutional Review Board and followed the tenets of the Declaration of Helsinki (1964). The healthy participants in this study comprised subjects who had visited the Armed Forces Capital Hospital (AFCH) for a health screening checkup. The subjects who met the eligibility criteria provided written informed consent to participate.

### Study Subjects

The subjects underwent a comprehensive ophthalmic examination, including visual acuity assessment, slit-lamp biomicroscopy, gonioscopy, Goldmann applanation tonometry, refraction, and dilated fundus examination. Additionally, central corneal thickness (CCT) (NT-530, NIDEK Co., LTD, Gamagori, Aichi, Japan) and axial length (AXL) (IOLMaster, Carl Zeiss Meditec, Inc.) were measured. IOP was measured twice, once before standardized Valsalva maneuver and then during.

The present study included eyes with no glaucomatous optic disc changes (e.g., notching, rim thinning, retinal nerve fiber layer [RNFL] defect) on fundus examination as well as eyes within the normal ranges for peripapillary RNFL thicknesses. The excluded subjects were those with (1) a history of ocular trauma, (2) a history of systemic or ocular infection, (3) a history of systemic disease (i.e., hypertension or diabetes), (4) a history of any intraocular surgery including cataract surgery, (5) IOP >21 mmHg, or (6) poor cooperation (making performance of standardized Valsalva maneuver with a sustained minimum expiratory pressure of 30 mmHg impossible). Only the right eye was selected for the analysis.

The study subjects were categorized into five groups by age: group A (age 20 to 29, *n* = 15), group B (age 30 to 39, *n* = 14), group C (age 40 to 49, *n* = 13), group D (age 50 to 59, *n* = 12), and group E (age 60 and over, *n* = 9).

### Protocol for the Standardized Valsalva Maneuver

**Figure 1:**
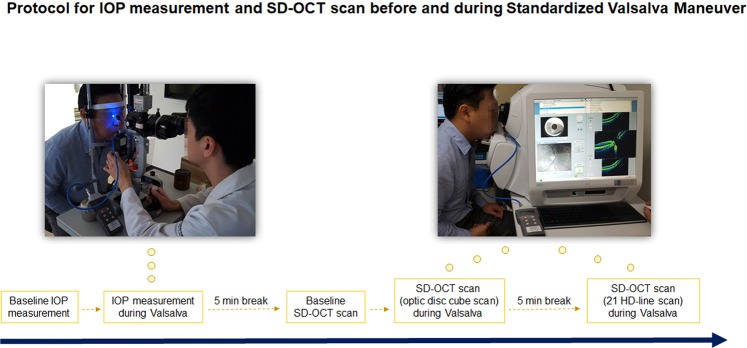
Protocol for intraocular pressure (IOP) measurement and spectral-domain optical coherence tomography (SD-OCT) scanning before and during the standardized Valsalva maneuver.

The detailed method for performance of the standardized Valsalva maneuver has been reported elsewhere^26^. The subjects were asked to exhale into a mouthpiece connected to a handheld differential pressure meter (OMEGA HHP-801TM; OMEGA Engineering Inc., Connecticut, USA). They were instructed to maintain the expiratory pressure at a minimal level of 30 mmHg for over 15 seconds. The expiratory pressure during the Valsalva maneuver was recorded at least 15 seconds after initiation of the standardized Valsalva maneuver. The expiratory pressures were monitored during IOP measurements or OCT scans, and the examiner encouraged the patients to maintain their expiratory pressure at a minimal level of 30 mmHg. The subject who cannot maintain his/her expiratory pressure at a minimal level of 30 mmHg were excluded from the study. The agreements for the expiratory pressures during IOP measurement and OCT scans were evaluated by Bland-Altman plot. The expiratory pressures according to the age groups (younger [groups A to C] vs. older [groups D and E]) were compared. After each Valsalva maneuver, the subjects were directed to take a five-minute break. The entire protocol for the analysis is outlined in Fig. 1 (written informed consent provided for publication of identifying information/images in an online open-access publication).

### EDI SD-OCT Imaging

Optic disc scanning was performed using the Cirrus HD-OCT 5000 (Carl Zeiss Meditec, Inc., Dublin, CA, USA) based on the following protocol: 200 × 200 optic disc cube scan and 21-HD line scans (9 mm length, enhanced depth imaging [EDI] mode) centered on the optic disc. All of the scans were performed with the subject in a sitting position, first as a baseline, and then with the subject sustaining the standardized Valsalva maneuver. The average peripapillary RNFL thickness and the following ONH parameters were automatically measured with the built-in analysis algorithm (software version 6.0; Carl Zeiss Meditec): rim area, disc area, average cup-to-disc ratio (C/D), vertical C/D, and cup volume.

### Measurement of LC Depth and BMO Distance

To enhance LC visibility, adaptive compensation was performed on all of the optic disc scan images according to the relevant protocol (contrast exponent = 2, threshold exponent = 6)^28,29^. All of the measurements were performed using ImageJ software (developed by Wayne Rasband, National Institutes of Health, Bethesda, MD, USA; available at http://imagej.nih.gov/ij/). The scaling of the acquired image was corrected to 1:1 prior to the measurement. The maximal LC depth was defined as the maximal vertical distance between the reference plane connecting Bruch’s membrane opening (BMO) and the anterior LC surface. The area enclosed by the visible anterior LC surface, the BMO reference plane, and two vertical lines connecting the two planes were measured. The mean LC depth was computed by dividing this area by the length between the two vertical lines. The BMO distance was defined as the distance between the two terminations of the BMO. The measurement was performed and averaged from the central three of the optic disc scans, in both scans (before and during Valsalva challenge). Excellent interobserver reproducibility for LC depth measurement has been reported elsewhere^8,9,26^.

To compare the visibility of the anterior LC among the age groups, the LC visibility was graded as reported by Girard *et al*.^30^. The anterior LC was graded as 0 if no part of it was visible between the two BMO points, 1 if less than 25% of the width was visible, 2 for 25 to 50%, 3 for 50 to 75%, and 4 for greater than 75%. The measurement was performed by an experienced ophthalmologist (Y.W.K.) who was masked to each of the subjects’ clinical information. The OCT scans with signal strength <6 and/or with visibility of the anterior LC surface less than 25% of the width between the two BMO points were excluded from the analysis.

### Data Analysis

The LC displacement during the standardized Valsalva maneuver was deemed to be statistically significant when it exceeded 1.96-times the intersession standard deviation, which had been determined to be 22.5 μm^26^. The continuous variables among the five age groups were compared by one-way analysis of variance (ANOVA) with Scheffe’s post hoc analysis. The categorical variables were compared using a chi-square test. The measurement values before and during the standardized Valsalva maneuver were compared using a paired t-test. The generalized linear model was used to investigate the influence of several factors (age, sex, IOP, IOP change and expiratory pressure during standardized Valsalva maneuver, AXL, CCT, RNFL thickness, rim area, disc area, average C/D, cup volume, baseline LC depth, and baseline BMO distance) on the degree of LC displacement during the standardized Valsalva maneuver, first with a univariate model and then with a multivariable model that included variables from the univariate model with *P* < 0.10. A negative value of LC depth change was regarded as anterior displacement, whereas a positive value was regarded as posterior displacement. The open-platform R software was used for the analysis^31^. Except where stated otherwise, the data are presented as mean ± standard deviations, and the level of statistical significance was set at *P* < 0.05.

## Results

### Baseline Characteristics

A total of 70 healthy volunteers were initially recruited. Five subjects were excluded due to unclear visibility of the anterior LC surface (less than 25% of width between the two BMO points was visible), and two others (aged over 60) owing to difficulties in performing the standardized Valsalva maneuver (expiratory pressure less than 30 mmHg). Finally, 63 healthy volunteers were included in the present study. Table 1 provides the summarized demographics of the participants. Younger eyes (groups A and B) tended to be more myopic and to have smaller disc area, average and vertical C/D, and cup volume than older eyes (group E).

**Table 1:** Subject Demographics.

Variables	Age groups (*n* = 63)					*P*-value	Post-hoc analysis
	Group A (age 20–29, *n* = 15)	Group B (age 30–39, *n* = 14)	Group C (age 40–49, *n* = 13)	Group D (age 50–59, *n* = 12)	Group E (age ≥ 60, *n* = 9)		
Gender, female, *n*(%)	3 (20.0)	6 (42.9)	4 (30.8)	1 (8.3)	6 (66.7)	0.046^†^	—
SE, *D*	−**2**.**44** ± **2**.**18**	−**1**.**78** ± **2**.**31**	−**0**.**98** ± **1**.**43**	−**0.45** ± **1**.**29**	−**0**.**28** ± **1**.**27**	**0**.020*	**A** < **D**,**E**
AXL, mm	**25**.**01** ± **0.96**	**25**.**30** ± **1**.**14**	**23**.**98** ± **0**.**89**	**23**.**55** ± **0**.**74**	**24**.**02** ± **0**.**88**	<**0**.001*	**A** > **C**,**D**, **B** > **C**,**D**,**E**
CCT, μm	537.3 ± 40.7	539.1 ± 39.9	548.2 ± 40.8	555.1 ± 44.0	540.2 ± 37.7	0.71*	
RNFL thickness, μm	94.7 ± 10.3	95.6 ± 8.9	97.1 ± 5.7	93.1 ± 11.1	89.8 ± 8.8	0.43*	
Rim area, mm^2^	1.23 ± 0.20	1.22 ± 0.17	1.33 ± 0.19	1.27 ± 0.19	1.18 ± 0.25	0.41*	
Disc area, mm^2^	**1**.**60** ± **0.28**	**1**.**66** ± **0**.**30**	**2**.**03** ± **0**.**42**	**1**.**95** ± **0**.**35**	**2**.**20** ± **0**.**51**	**0**.001*	**A** < **C**,**E**, **B** < **E**
Average C/D	**0**.**44** ± **0**.**17**	**0**.**50** ± **0**.**07**	**0**.**56** ± **0**.**10**	**0**.**55** ± **0**.**15**	**0**.**65** ± **0**.**12**	**0**.003*	**A**,**B** < **E**
Vertical C/D	**0**.**40** ± **0**.**16**	**0**.**46** ± **0**.**08**	**0**.**52** ± **0**.**09**	**0**.**51** ± **0**.**15**	**0**.**61** ± **0**.**09**	**0**.002*	**A**,**B** < **E**
Cup volume, mm^3^	**0**.**138** ± **0**.**149**	**0**.**126** ± **0**.**102**	**0**.**209** ± **0**.**151**	**0**.**194** ± **0**.**149**	**0**.**348** ± **0**.**185**	**0**.008*	**A**,**B** < **E**
LC visibility score	2.47 ± 0.52	2.57 ± 0.51	2.85 ± 0.69	2.33 ± 0.49	2.89 ± 0.93	0.16*	

Mean ± standard deviation, statistically significant values are shown in bold. *Comparison was performed using one-way analysis of variance with post hoc Scheffe’s multiple comparison testing. ^†^Comparison was performed using chi-square test. SE: spherical equivalence, AXL: axial length, CCT: central corneal thickness, RNFL: retinal nerve fiber layer, C/D: cup-to-disc ratio.

### Expiratory Pressure During Standardized Valsalva Maneuver

The expiratory pressure ranged from 30 to 56 mmHg during IOP measurement, 30 to 54 mmHg during optic disc cube scan, and 30 to 55 mmHg during 21 HD-line scan. The expiratory pressure during IOP measurement and OCT scans revealed right-skewed distributions (Supplementary Fig. S1). The greatest expiratory pressure value was obtained from a 43 year-old male volunteer.

**Figure 2:**
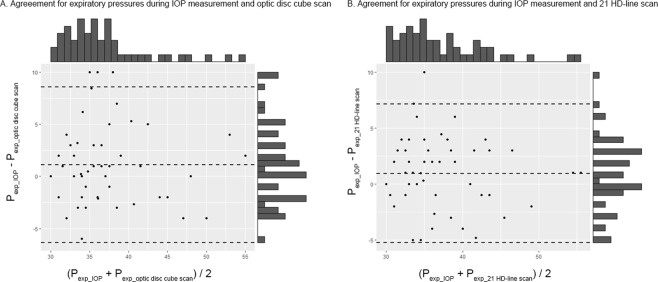
Bland-Altman plot and marginal histogram demonstrating agreement and distribution of expiratory pressure during the standardized Valsalva maneuver. Agreements for (**A**) expiratory pressure during intraocular pressure (IOP) measurement and optic disc cube scan and (**B**) expiratory pressure during IOP measurement and 21-HD line scan.

There were no significant differences in expiratory pressures during IOP measurement (37.2 ± 5.8 vs. 38.0 ± 6.2 mmHg, *P* = 0.61), optic disc cube scan (36.3 ± 5.8 vs. 36.5 ± 6.2 mmHg, *P* = 0.89), or 21 HD-line scan (36.0 ± 6.0 vs. 37.3 ± 6.4 mmHg, *P* = 0.46) between younger (groups A to C, *n* = 33) and older age groups (groups D and E, *n* = 21). There were no significant correlations between age and expiratory pressure for the standardized Valsalva maneuver during IOP measurement (ρ = 0.09, *P* = 0.52), optic disc cube scan (ρ = −0.02, *P* = 0.89) or 21-HD line scan (ρ = 0.12, *P* = 0.40). Bland-Altman plots between expiratory pressure during IOP measurement and optic disc cube scans as well as 21-HD line scans and their marginal histograms are provided in Fig. 2.

### IOP Change During the Standardized Valsalva Maneuver

The resting-state IOP significantly increased during the standardized Valsalva maneuver, from 13.2 ± 2.9 mmHg to 18.6 ± 5.2 mmHg (*P* < 0.001). This increase was evident in 60 of 63 eyes (95.2%). The IOP of three eyes (4.8%), including one eye from group B and two eyes from group E, decreased 2, 2, and 1 mmHg, respectively.

### ONH and LC Parameter Change During the Standardized Valsalva Maneuver

There was no significant change of RNFL thickness (94.4 ± 9.2 μm vs. 94.2 ± 9.5 μm) or optic nerve head parameters during the standardized Valsalva maneuver (Table 2). The maximal (456.4 ± 113.7 μm) and mean LC depth (397.4 ± 100.4 μm) significantly decreased to 436.7 ± 109.9 μm (*P* < 0.001) and 386.0 ± 103.8 μm (*P* = 0.004), respectively. Twenty-seven (27) eyes (42.9%) showed significant anterior LC displacement, whereas three eyes (4.8%) revealed significant posterior LC displacement during the standardized Valsalva maneuver. The BMO distance did not change significantly during the standardized Valsalva maneuver (1520 ± 169.2 μm vs. 1520.8 ± 169.0 μm, *P* = 0.87).

**Table 2:** Changes of Variables during the Standardized Valsalva Maneuver.

	Baseline	During Valsalva	*P*-value*
**IOP, mmHg**	**13**.**2** ± **2**.**9**	**18**.**6** ± **5**.**2**	<**0**.**001**
**ONH parameters**			
RNFL thickness, μm	94.4 ± 9.2	94.2 ± 9.5	0.96
Rim area, mm^2^	1.25 ± 0.20	1.25 ± 0.20	0.72
Disc area, mm^2^	1.86 ± 0.42	1.85 ± 0.42	0.78
Average C/D	0.53 ± 0.14	0.53 ± 0.14	0.52
Vertical C/D	0.49 ± 0.14	0.49 ± 0.13	0.96
Cup volume, mm^3^	0.191 ± 0.159	0.190 ± 0.159	0.84
**LC parameters**			
Maximal LC depth, μm	**456**.**4** ± **113**.**7**	**436**.**7** ± **109**.**9**	<**0**.**001**
Mean LC depth, μm	**397**.**4** ± **100**.**4**	**386**.**0** ± **103**.**8**	**0**.**004**
BMO distance, μm	1520.0 ± 169.2	1520.8 ± 169.0	0.87

Mean ± standard deviation, statistically significant values are shown in bold. *Comparison performed using paired t-test. IOP: intraocular pressure, ONH: optic nerve head, LC: lamina cribrosa, BMO: Bruch’s membrane opening.

### LC Parameter Change According to Age

There were no significant differences in LC visibility among the age groups (*P* = 0.16). The proportion of significant anterior LC displacement during the standardized Valsalva maneuver tended to decrease with age (group A, 80.0%, group B, 71.4%, group C, 23.1%, group D, 0%, group E, 22.2%). Only eyes in groups C (*n* = 1) and D (*n* = 2) exhibited significant posterior LC displacement during the standardized Valsalva maneuver.

**Figure 3:**
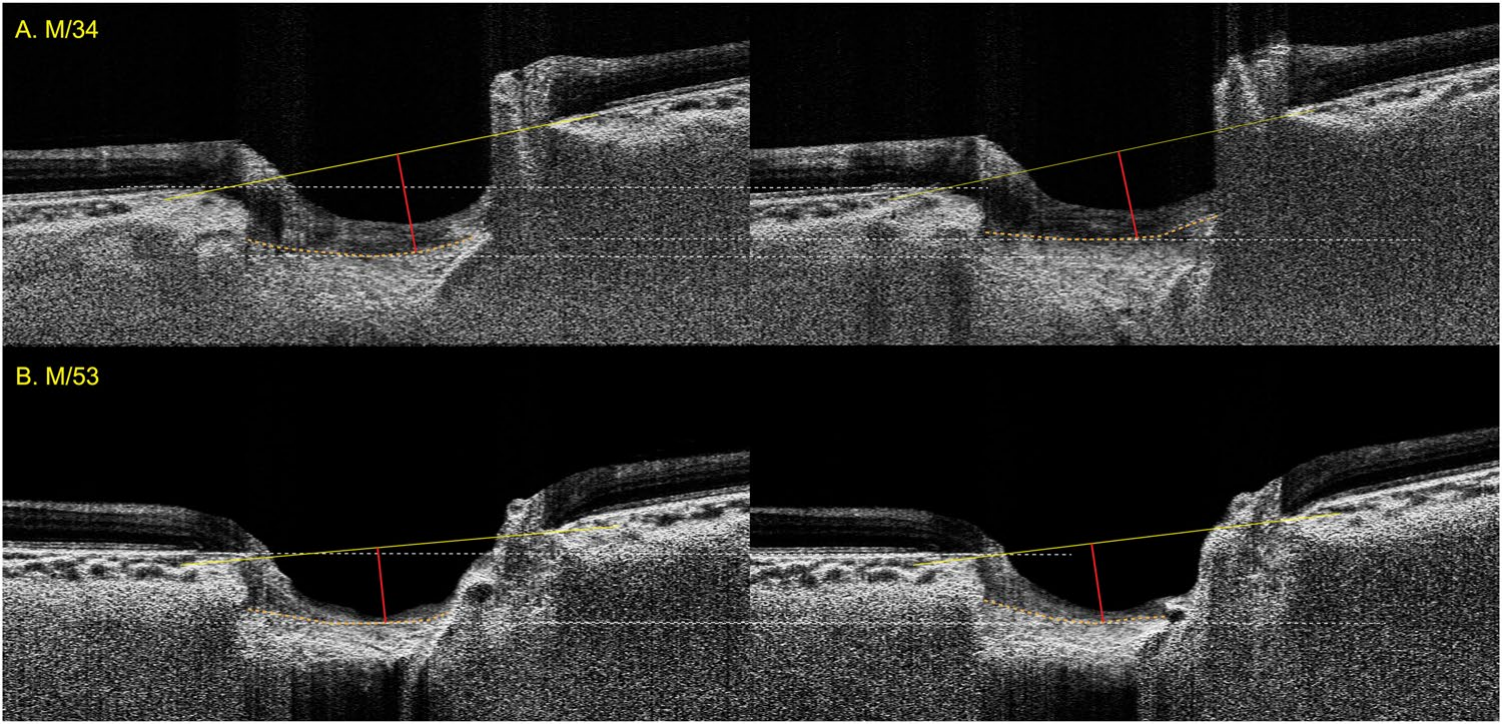
Age-dependent variation of lamina cribrosa (LC) displacement during the standardized Valsalva maneuver. (**A**) Enhanced depth imaging (EDI) optic disc scan of 34-year-old healthy male (AXL = 25.37 mm, CCT = 527 µm) at baseline (left) and during the standardized Valsalva maneuver (right). The IOP increased from 12 to 17 mmHg. The maximal LC depth decreased from 577 to 542 µm, and the mean LC depth decreased from 521.3 to 495.1 µm. (**B**) EDI optic disc scan of 53-year-old healthy male (AXL = 24.03 mm, CCT = 559 µm) showing maximal LC depth change from 292 to 315 µm and mean LC depth change from 286.5 to 278.5 µm during the standardized Valsalva maneuver, respectively. The IOP increased from 10 to 17 mmHg. The maximal LC depth has been depicted in red solid line.

The maximal LC depth significantly decreased during the standardized Valsalva maneuver in groups A (from 489.3 ± 131.5 μm to 454.3 ± 131.9 μm, *P* < 0.001), B (from 506.6 ± 122.4 μm to 470.3 ± 116.3 μm, P < 0.001), and C (from 408.2 ± 78.9 μm to 394.3 ± 87.4 μm, *P* = 0.021). However, eyes in groups D and E did not exhibit significant change (Table 3). The mean LC depth change was significant only in groups A (from 425.8 ± 105.3 μm to 398.7 ± 120.0 μm, *P* < 0.001) and B (from 434.2 ± 88.3 μm to 412.6 ± 98.6 μm, *P* = 0.017), but not in groups C to E. Representative cases are provided in Fig. 3.

**Table 3:** Lamina Cribrosa Depth Change during the Standardized Valsalva Maneuver According to Age.

	Maximal LC depth, μm			Mean LC depth, μm			BMO distance, μm		
	Baseline	During Valsalva	*P*-value*	Baseline	During Valsalva	*P*-value*	Baseline	During Valsalva	*P*-value*
Overall	**456**.**4** ± **113**.**7**	**436**.**7** ± **109**.**9**	**<0**.**001**	**397**.**4** ± **100**.**4**	**386**.**0** ± **103**.**8**	**0**.**004**	1520.0 ± 169.2	1520.8 ± 169.0	0.87
Age 20–29	**489**.**3** ± **131**.**5**	**454**.**3** ± **131**.**9**	**<0**.**001**	**425**.**8** ± **105**.**3**	**398**.**7** ± **120**.**0**	**<0**.**001**	1433.7 ± 131.2	1419.3 ± 128.7	0.07
Age 30–39	**506**.**6** ± **122**.**4**	**470**.**3** ± **116**.**3**	**<0**.**001**	**434**.**2** ± **88**.**3**	**412**.**6** ± **98**.**6**	**0**.**017**	1514.6 ± 121.8	1532.3 ± 119.1	0.24
Age 40–49	**408**.**2** ± **78**.**9**	**394**.**3** ± **87**.**4**	**0**.**021**	349.4 ± 92.6	342.5 ± 93.2	0.45	1564.8 ± 188.6	1549.0 ± 186.7	0.11
Age 50–59	435.6 ± 113.9	437.6 ± 111.6	0.69	388.6 ± 115.3	395.2 ± 113.4	0.49	1531.0 ± 146.1	1546.4 ± 148.6	0.22
Age ≥60	420.7 ± 77.8	414.9 ± 81.0	0.36	374.1 ± 80.6	374.3 ± 83.4	0.67	1592.7 ± 248.2	1597.4 ± 241.5	0.67

Mean ± standard deviation, statistically significant values are shown in bold. *Comparison performed using paired t-test. LC: lamina cribrosa, BMO: Bruch’s membrane opening.

**Figure 4:**
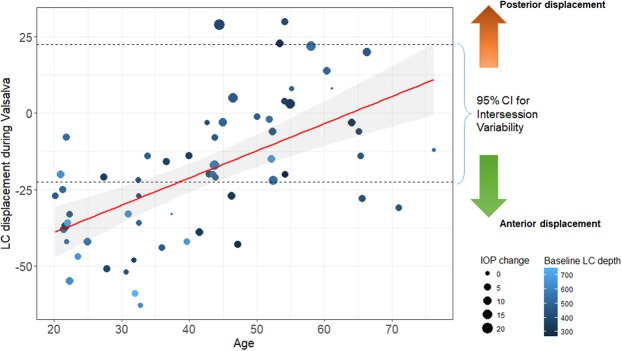
Correlation between age and maximal lamina cribrosa (LC) displacement during the standardized Valsalva maneuver. The dotted line represents the 95% confidence interval (CI) for intersession variability. LC displacement beyond this range was regarded as statistically significant. Age, intraocular pressure (IOP) change during the Valsalva challenge, and baseline maximal LC depth were all significantly associated with degree of LC displacement.

Both maximal and mean LC depth change during the Valsalva challenge revealed significant correlation with age (ρ = 0.60, *P* < 0.001 and ρ = 0.39, *P* = 0.002, respectively) (Fig. 4). The BMO distance change was not significant in any of the age groups during the standardized Valsalva maneuver (Table 3).

### Factors Associated with Anterior LC Displacement During the Standardized Valsalva Maneuver

In the univariate analysis, the factors associated with anterior LC displacement during the standardized Valsalva maneuver were younger age (*P* < 0.001), smaller IOP change during the standardized Valsalva maneuver (*P* = 0.001), greater AXL (*P* < 0.001), smaller disc area (*P* = 0.004), smaller average C/D (*P* = 0.001), smaller cup volume (*P* = 0.007), and greater baseline maximal LC depth (*P* = 0.036) (Table 4).

**Table 4:** Factors associated with Maximal Lamina Cribrosa Depth Change during the Standardized Valsalva Maneuver.

Variables	Univariate Model			Multivariable Model		
	β	SE	*P*-value	β	SE	*P*-value
Age, per yr	**0**.**896**	**0**.**153**	<**0**.**001**	**0**.**491**	**0**.**180**	**0**.**009**
IOP, per mmHg	0.053	1.011	0.96			
IOP change during Valsalva, per mmHg	**2**.**165**	**0**.**637**	**0**.**001**	**1**.**648**	**0**.**514**	**0**.**002**
Expiratory pressure during Valsalva, per mmHg	0.632	0.492	0.20			
Gender, female	1.656	6.054	0.79			
AXL, per mm	−8.494	2.237	<0.001	−3.642	2.172	0.10
CCT, per μm	0.039	0.079	0.62			
RNFLT, per μm	−0.187	0.309	0.55			
Rim area, per mm^2^	−9.943	14.326	0.49			
Disc area, per mm^2^	18.820	6.380	0.004	−11.357	8.014	0.16
Average C/D, per 1	62.339	18.060	0.001	33.309	26.814	0.22
Cup volume, per mm^3^	47.133	16.844	0.007	29.046	27.175	0.29
Baseline maximal LC depth, per μm	**−0**.**052**	**0**.**024**	**0**.**036**	−**0**.**058**	**0**.**022**	**0**.**013**
Baseline BMO distance, per μm	0.017	0.017	0.32			

Statistical analysis was performed using the generalized linear model. Statistically significant values are shown in bold. *Factors with *P* < 0.10 in the univariate analysis were included in the multivariable analysis.

In the multivariable model, younger age (β = 0.491, *P* = 0.009), smaller IOP change during the standardized Valsalva maneuver (β = 1.648, *P* = 0.002), and greater baseline maximal LC depth (β = −0.058, *P* = 0.013) were significantly associated with anterior LC displacement during the standardized Valsalva maneuver.

## Discussion

The present study demonstrated age-dependent variation of LC displacement during the standardized Valsalva maneuver. Younger eyes showed significant anterior LC displacement, while older eyes revealed equivocal displacement during the standardized Valsalva maneuver. This might have been originated from the age-related differences of the LC and peripapillary sclera.

Valsalva maneuver can induce an increase of both IOP and CSFP^32–34^. Elevated intrathoracic pressure during the Valsalva challenge can reduce venous return via the superior and inferior vena cava, leading to engorgement of the distal venous system and, thus, increased IOP and CSFP. Zhang *et al*.^25^ recently investigated IOP and CSFP changes during the Valsalva challenge in patients with lumbar puncture. They reported that the CSFP increment (10.5 ± 2.7 mmHg) exceeded that of IOP (1.9 ± 2.4 mmHg), with the consequence that the TLPD was decreased or reversed. Their subsequent analysis revealed inward movement of ONH parameters (decreased C/D, cup volume, and depth, with increased neuroretinal rim volume and RNFL thickness) during the Valsalva challenge. In this light, our group demonstrated anterior displacement of the LC during the standardized Valsalva maneuver in young healthy eyes^26^. We found no significant change in choroidal thickness or neural canal opening diameter. Previous studies also reported no significant change in choroidal thickness during the Valsalva maneuver in both young^35^ and older^27^ subjects. This suggests that anterior LC displacement might not originate from choroidal thickness change or stretching by scleral expansion due to IOP elevation.

The LC and peripapillary sclera stiffen with age^36–39^. This is a natural consequence of aging, whereby collagen and the extracellular matrix (ECM) undergo a process of dynamic remodeling. During normal aging, the type of collagen, elastin content, and proteoglycans change, and the amount of ECM increases^36,40,41^. The crosslinking of collagen and elastin increases with the accumulation of advanced glycation end products. Girard *et al*.^42^ investigated age-related differences in the biomechanical properties of the sclera in monkey eyes, and demonstrated significantly stiffer posterior sclera in old monkeys. Studies based on human sclera, meanwhile, have shown that the sclera in older subjects was thinner and had smaller strain for a given level of IOP stress^38,39^. Age-related stiffening of the sclera differs by race, eyes of subjects of African descent having shown more rapid stiffening with age, a higher shear modulus and a smaller collagen fibril crimp angle compared with those of subjects of European descent^15,16^. This might induce racial differences of LC depth change in response to acute IOP elevation^17^.

Taken together, the mechanical response of the LC is very complex; as such, tissue properties as well as IOP and CSFP need to be given due consideration. The present study demonstrated that younger age, a smaller increase of IOP during the Valsalva challenge and greater baseline maximal LC depth were associated with anterior LC displacement during the standardized Valsalva maneuver. This finding is quite analogous to Lee *et al*.’s findings^43^, which showed a reversal of LC displacement after IOP reduction by trabeculectomy. In that study, moreover, the magnitude of LC reversal was significantly associated with younger age, greater percent-IOP reduction, and greater preoperative LC displacement. In both that study and ours, the stiffness of the LC and peripapillary sclera as well as the pressure difference at the LC boundary might have influenced LC behavior. In this regard, detection of LC displacement during the Valsalva maneuver may be an indirect way to measure LC stiffness in a noninvasive manner.

The present study showed no significant differences in expiratory pressure during the standardized Valsalva maneuver according to the age groups. It may be argued that the potential differences in the ability for performing the Valsalva maneuver with age was not observed in the present study. Subjects were instructed to maintain the expiratory pressure at a minimal level of 30 mmHg for over 15 seconds during the standardized Valsalva maneuver. This criteria (expiratory pressure 30 mmHg) may not be the maximal exertional state for younger subjects. Although there might have been substantial age-related difference in the maximal intensity of the Valsalva maneuver, our criteria may not have had reached to this state.

Anterior LC displacement during the Valsalva challenge does not imply that performance of the Valsalva maneuver can be protective against glaucoma development. Posterior displacement of the LC in glaucomatous eyes is the result of irreversible LC remodeling from chronic exposure to IOP stress. The findings from the present study only reveal transient LC behavior, which can be highly dependent on the stiffness of the LC and peripapillary sclera. Schuman *et al*.^44^ warned of the risk of performing the Valsalva maneuver, having demonstrating increased IOP and visual field defects in professional musicians who play high-resistance wind instruments. The LC can be in a more stressful environment as IOP and CSFP increase during the Valsalva maneuver, which squeezes the LC and stimulates remodeling of LC cells. Altogether, frequent and cumulative stresses induced by the Valsalva maneuver might not be beneficial to LC physiology. The issue, in any case, requires further investigation.

One may argue that anterior LC displacement during the Valsalva maneuver originates from IOP-induced scleral canal expansion that pulls the LC taut^17,45,46^. However, the present study found no change in BMO distance during the Valsalva challenge, either overall or by age group. The pressure dynamics on the LC in the present study differ from those in other studies that investigated LC depth change during acute IOP elevation, possibly because CSFP, as well as IOP, is elevated during the Valsalva maneuver. This could explain why the expansion of neural canal openings (or increased BMO distance) was not prominent in the present study.

The present study has the following shortcomings. First, LC visibility was limited, so that the mean LC visibility grade was only 2.6 ± 0.6 (i.e., nearly 50% of the entire LC was visible). This possibly was due to shadowing from thicker neuroretinal rim tissues and vessels in healthy eyes. The nasal region of the LC was difficult to investigate, and so measurement of mean LC depth most often included the temporal region. However, there was no significant difference in the LC-visibility grade according to age; thus, the measurement might not have been biased from variable LC visibility with age. Second, the LC was not evaluated in a three-dimensional manner but only from the central section of the LC. This was also due to the limited visibility of the LC with thick neuroretinal rims in healthy eyes. The present study might not have fully captured LC change during the Valsalva maneuver. Third, effect of Valsalva-induced cardiovascular changes, including blood pressure or heart rate, were not considered in the present study. Age-dependent differences in these factors may have influenced the findings of the present study. Fourth, all the study participants were Koreans. As the tissue properties of the LC and sclera vary by race, our results cannot be generalized to other ethnicities. Finally, the present methodology cannot provide any information on actual tissue stiffness of LC. Further histological *ex-vivo* studies may expand our findings.

In conclusion, LC displacement during the standardized Valsalva maneuver varied with age and IOP change. The present data can enhance our understanding of the LC in the biomechanical perspective, specifically in that age-related tissue properties as well as translaminar pressure dynamics are crucial to LC strain.

## Supplementary information

Supplementary Figure 1

## Data Availability

The datasets generated during and/or analysed during the current study are available from the corresponding author on reasonable request.
