# Supplementary material for: Age-Dependent Variation of Lamina Cribrosa Displacement During the Standardized Valsalva Maneuver
Source: Sci Rep. 2019 Apr 30;9:6645. doi: 10.1038/s41598-019-43206-6 (PMC6491605; doi:10.1038/s41598-019-43206-6)
Supplement: Supplementary file 1 — Supplementary Figure 1 [file 41598_2019_43206_MOESM1_ESM.pdf]

Supplementary Information

Age-Dependent Variation of Lamina Cribrosa Displacement During the Standardized Valsalva Maneuver

Yong Woo Kim, MD[1,2], Dong Hyun Lee, MD[2], Hyung Bin Lim, MD[2], Baek-Lok Oh, MD[1,2], Young Kook Kim, MD[1], Michael J. A. Girard, PhD[3,4], Jean Martial Mari, PhD[5], Ki Ho Park, MD, PhD[1], Jin Wook Jeoung, MD, PhD[1]

[1]Department of Ophthalmology, Seoul National University Hospital, Seoul National University College of Medicine, Seoul, Korea

[2]Department of Ophthalmology, Armed Forces Capital Hospital, Seongnam, Korea

[3]Department of Biomedical Engineering, National University of Singapore, Singapore

[4]Singapore Eye Research Institute, Singapore

[5]University of French Polynesia, Tahiti, French Polynesia

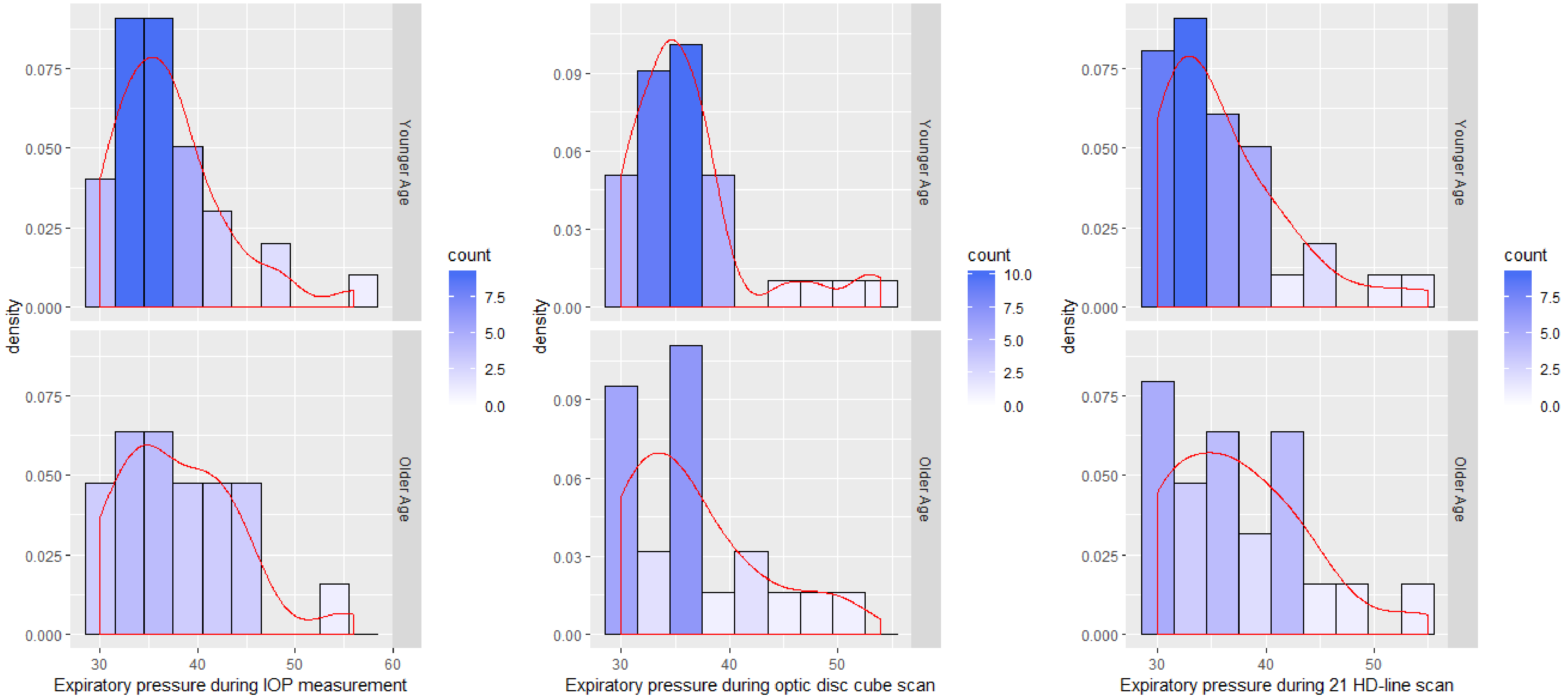

density
count
Younger Age
Older Age
Expiratory pressure during IOP measurement
Expiratory pressure during optic disc cube scan
Expiratory pressure during 21 HD-line scan

Supplementary Figure S1. Distribution of expiratory pressures during IOP measurement, optic disc cube scan and 21 HD-lines scan between younger and older age groups.

The expiratory pressure during IOP measurement and OCT scans revealed right-skewed distributions. There were no significant differences in expiratory pressures during IOP measurement (*P*= 0.61), optic disc cube scan (*P*= 0.89), or 21 HD-line scan (*P*= 0.46) between younger (groups A to C, *n* = 33) and older age groups (groups D and E, *n* = 21).
